# High Electrochemiluminescence from Ru(bpy)_3_^2+^ Embedded Metal–Organic Frameworks to Visualize Single Molecule Movement at the Cellular Membrane

**DOI:** 10.1002/advs.202204715

**Published:** 2022-11-03

**Authors:** Binxiao Li, Xuedong Huang, Yanwei Lu, Zihui Fan, Bin Li, Dechen Jiang, Neso Sojic, Baohong Liu

**Affiliations:** ^1^ Department of Chemistry Shanghai Stomatological Hospital State Key Laboratory of Molecular Engineering of Polymers Fudan University Shanghai 200433 China; ^2^ State Key Laboratory of Analytical Chemistry for Life and School of Chemistry and Chemical Engineering Nanjing University Nanjing Jiangsu 210093 China; ^3^ Bordeaux INP Institute of Molecular Science (ISM), and CNRS UMR 5255 University of Bordeaux Pessac 33607 France

**Keywords:** dynamic tracking, metal–organic frameworks, nanoconfined‐enhanced emission, nanoemitter, single‐molecule ECL

## Abstract

Direct imaging of single‐molecule and its movement is of fundamental importance in biology, but challenging. Herein, aided by the nanoconfinement effect and resultant high reaction activity within metal–organic frameworks (MOFs), the designed Ru(bpy)_3_^2+^ embedded MOF complex (RuMOFs) exhibits bright electrochemiluminescence (ECL) emission permitting high‐quality imaging of ECL events at single molecule level. By labeling individual proteins of living cells with single RuMOFs, the distribution of membrane tyrosine‐protein‐kinase‐like7 (PTK7) proteins at low‐expressing cells is imaged via ECL. More importantly, the efficient capture of ECL photons generated inside the MOFs results in a stable ECL emission up to 1 h, allowing the in operando visualization of protein movements at the cellular membrane. As compared with the fluorescence observation, near‐zero ECL background surrounding the target protein with the ECL emitter gives a better contrast for the dynamic imaging of discrete protein movement. This achievement of single molecule ECL dynamic imaging using RuMOFs will provide a more effective nanoemitter to observe the distribution and motion of individual proteins at living cells.

## Graphical abstract

Ru(bpy)_3_^2+^ in single nanoporous metal–organic frameworks complex (RuMOFs) is designed as novel nanoemitter with high electrochemiluminescence (ECL), which permits the collection of discrete ECL events at single molecules level. Near‐zero ECL background surrounds the target labeled with the confinement‐enhanced emitter, achieving unprecedentedly the visualized dynamic characteristics monitoring of individual proteins at different living cells surface.

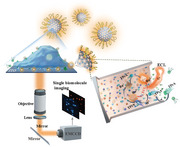

## 1 Introduction

As the basic component of cellular plasma membrane, proteins play an essential role to maintain cellular structure and metabolism. The current studies focus on the determination of their distribution at the cells. However, tracking their movement and identifying their intended destinations are challenging because it requires the operando observation of individual proteins at living cell.^[^ ^1^, ^2^ ^]^ Recently, single‐molecule photoluminescence (PL) imaging has emerged as the tool for the research of protein movements, revealing stochastic processes of proteins.^[^ ^1^, ^3^ ^]^ However, the limitations in PL‐based imaging, including irreversible photobleaching and strong luminescence background, interfere with the dynamic observation of single protein movements. ECL refers to the optical radiation process caused by energy relaxation of the excited state of a luminophore and photon emission after an electrochemical reaction.^[^ ^4^ ^]^ By contrast with PL, ECL does not rely on external light source and thus it minimizes the photobleaching and interference of background to the quantification. Accordingly, the quality (mainly signal‐to‐noise) of ECL imaging is improved to significantly enhance the accuracy of protein visualization on cells.^[^ ^5^, ^6^ ^]^ Despite these tremendous achievements, ECL microscopy of single proteins on living cells remains a challenging task due to the weak signal of the luminophore.

Previously, our team incorporated Ru(bpy)_3_^2+^ into silica/Au nanoparticles to prepare highly effective ECL nanoemitters (RuDSN/AuNPs).^[^ ^7^ ^]^ The further linkage of nanoemitter‐labeled antibody with the corresponding proteins at the surface of a fixed cell realizes the ECL visualization at single molecule level on cells. Despite the significant enhancement, in the ECL emission, the access of the reaction intermediates to Ru(bpy)_3_^2+^ inside RuDSN/AuNPs is still limited.^[^ ^8^, ^9^ ^]^ ≈30 000 molecules of Ru(bpy)_3_^2+^ were required to be embedded in one nanoemitter for the visualization of a single protein.^[^ ^7^ ^]^ Moreover, serious leaking of Ru(bpy)_3_^2+^ from these doped‐structure occurred leading to an obvious drop in the luminescence intensity during the continuous imaging. Therefore, it is difficult to apply these nanoemitters to dynamically visualize single molecules at the living cells. More robust ECL emitters with strong and stable ECL emission from a few Ru(bpy)_3_^2+^ molecules need to be further explored for the continuous study of single living cells.

Currently, various types of porous nanomaterials with well‐confined spaces are reported in the fields of controlled release,^[^ ^10^ ^]^ molecules/energy storage,^[^ ^11^ ^]^ and catalysis.^[^ ^12^ ^]^ By contrast with dye‐doped nanoparticles,^[^ ^13^ ^]^ it is inferred that the confined‐stable pore/channel structure can favor the embedment of dyes inside the nanomaterials. More importantly, the reaction intermediates could be retained inside the pores to promote the reaction rate.^[^ ^5^, ^14^, ^16^ ^]^ As an important member of porous materials, MOF materials are more prominent in terms of adjustable porosities and tailorable chemistry in addition to the above functions.^[^ ^17^ ^]^ In particular, MOF‐based composites and derivatives exhibit excellent enhanced electrochemical properties.^[^ ^17^, ^18^ ^]^ However, their related ECL imaging studies have not been reported.

Thus, the features of confinement‐enhanced MOF nanomaterials should be in line with our quest to enhance the ECL signals for individual cell study.

**Scheme 1:**
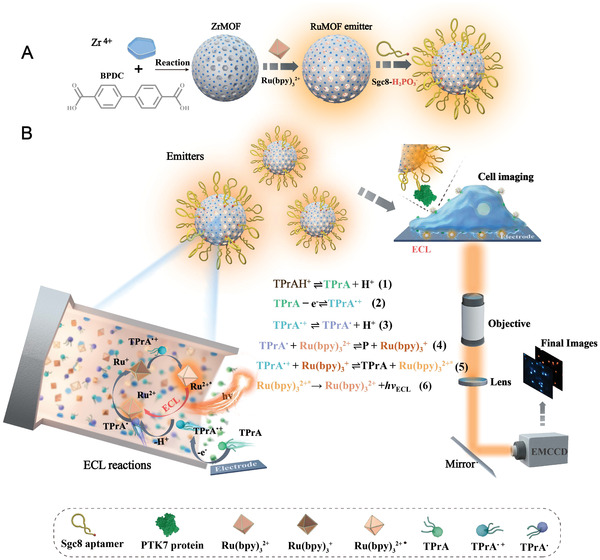
Schematic illustration of ECL nanoemitters synthesis and ECL imaging. A) Synthesis scheme of the RuMOFs via a facile solvothermal reaction. B) Imaging setup and mechanism with TPrA coreactant for the ECL visualization of single proteins at living cells.

Inspired by the confinement effect in electrochemistry,^[^ ^15^, ^19^ ^]^ Ru(bpy)_3_^2+^ molecules are assembled inside the Zr‐based MOF (ZrMOF) with the porous/channel structure to form RuMOFs as single ECL nanoemitter. We targeted PTK7 protein, which is a model transmembrane protein to transduce extracellular signals across the cell membrane. This receptor regulates several processes in embryonic development and tissue homeostasis including the establishment of cell polarity, the regulation of cell movement and migration. The specific aptamer for the PTK7 protein, phosphate‐terminal Sgc8 aptamers, decorates the surface of RuMOFs to construct the ECL nanoemitters (**Scheme** **1**A). Cells were grown on the electrode surface and then incubated with the RuMOFs. Upon imposing a sufficient potential in presence of the sacrificial tripropylamine (TPrA) co‐reactant, the ECL emission resulting from such nanoemitters is collected by ECL microscopy for the visualization of the PTK7 proteins and their movements (Scheme 1B; and Figure S1, Supporting Information).

## 2 Results and Discussion

**Figure 1:**
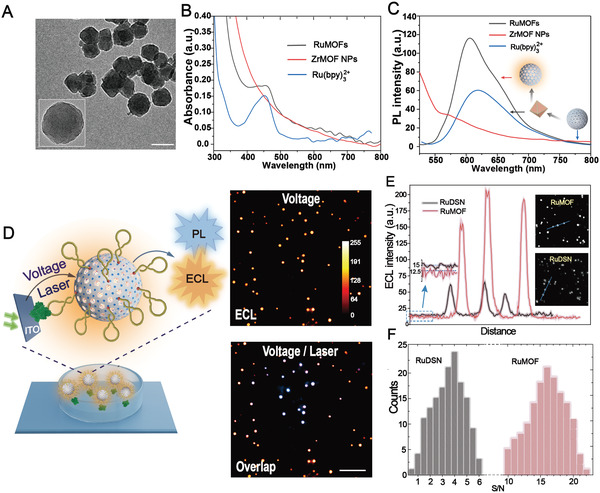
ECL performance characterization of RuMOFs nanoemitter. A) TEM image of RuMOFs and ZrMOF NPs (inset). Scale bar, 100 nm. B,C) UV–vis absorption spectra and photoluminescence spectra of RuMOFs, ZrMOF NPs, and Ru(bpy)_3_^2+^, respectively. D) Single‐molecule ECL and PL images of nanoemitters recorded with a voltage or laser. Exposure time is 1 s. Scale bars (white), 3 µm. E) ECL spatial‐intensity profiles of individual RuMOFs and RuDSN along blue line in ECL images (insets on the right). The background intensities are shown in the inset (left). F) ECL signal‐to‐noise (S/N) distribution of RuMOFs and RuDSN, respectively. The S/N of the RuDSN and RuMOF were integrated in units of 0.5 and 1, respectively. At least 150 nanoemitters were analyzed.

The transmission electron microscope (TEM) images show that RuMOFs present a porous nanostructure with spheroidal shape (**Figure** **1**A). The average diameter is about 90 nm, which is consistent with the dynamic light scattering (DLS) characterization (Figure S2, Supporting Information). TEM energy mapping results reveal that Ru is uniformly distributed in RuMOFs, similar to these elements (e.g., carbon, oxygen, nitrogen, and zirconium) in MOF framework (Figure S3, Supporting Information). Furthermore, compared with pure ZrMOF NPs, the zeta potential of RuMOFs does not change significantly (Figure S4, Supporting Information), excluding the possibility of the adsorption of Ru complexes at the ZrMOF NPs surface. Accordingly, the solution containing RuMOFs appears colorless and transparent (Figure S5, Supporting Information). Considering two polyhedral cavities with sizes of 23 and 12 Å in ZrMOF NPs,^[^ ^20^ ^]^ the spherical Ru(bpy)_3_^2+^ molecules may be reasonably accommodated inside the cavities of ZrMOF NPs.^[^ ^21^ ^]^

The absorption spectra of RuMOFs (Figure 1B) show a distinct absorption peak at ≈400–500 nm. This peak is characteristic of the metal‐to‐ligand charge transfer (MLCT) band^[^ ^22^, ^23^ ^]^ from the Ru(bpy)_3_^2+^ complexes incorporated into the MOF structure. Moreover, compared with Ru(bpy)_3_^2+^ complexes, the absorption peaks of RuMOFs have a slight redshift, exhibiting the distortion of the bipyridine ligands due to steric constraints.^[^ ^22^, ^24^ ^]^

As a result, the rigid‐pore framework and covalent interaction limits this distortion, resulting in a decrease in MLCT (d→π*) efficiency. The emission spectra of the pure ZrMOF NPs (Figure 1C) illustrates negligible PL emission that reflects the inertness of ZrMOF NPs to the excitation light. Contrary, the RuMOFs show a clear emission peak at 609 nm, which presents a 9 nm hypsochromic shift from the peak (618 nm) of the molecular Ru(bpy)_3_^2+^ complexes.

The peak intensity from RuMOFs is nearly twice than that from the molecular Ru(bpy)_3_^2+^ complexes. This increase is ascribed to the efficient retention of Ru(bpy)_3_^2+^ inside the ZrMOF NPs structure avoiding aggregation‐induced quenching. In addition, the nanosized confined space in RuMOFs restricts the movement or rotation of Ru(bpy)_3_^2+^. This effect increases the rigidity of the luminescent complex, and reduces the collision probability and energy loss caused by thermal motion. The strong interaction between Ru(bpy)_3_^2+^ and the surrounding MOF structure guarantees the negligible leakage of Ru(bpy)_3_^2+^ from RuMOFs, leading to a good stability in the emission for 7 days (Figure S6, Supporting Information).

The main goals of this work were to demonstrate the capability of our ECL probe for the dynamic imaging of single membrane proteins on living cells. In a first step, the aptamer modified RuMOFs nanoemitters were captured by immobilized PTK7 proteins at the electrode surface. In this configuration, clear ECL signal spots appear (Figure 1D) under the application of a voltage of 1.35 V. To ensure the ECL emission from the nanoemitters, a laser is applied transiently to collect the PL emission. The PL and ECL signal spots match well supporting the ECL emission from the nanoemitters in the image (Figure 1D). The ECL intensities from individual spots are close, suggesting the uniform ECL luminescence from these nanoemitters (Figure S7, Supporting Information). To further study the homogeneity in ECL emission, the ECL intensity from 600 emitters are measured. The statistical analysis illustrates that the emitter ECL signals concentrate in a limited high‐intensity range, exhibiting ideal ECL luminescence performance to ensure the adequacy of subsequent imaging (Figure S8, Supporting Information).

**Figure 2:**
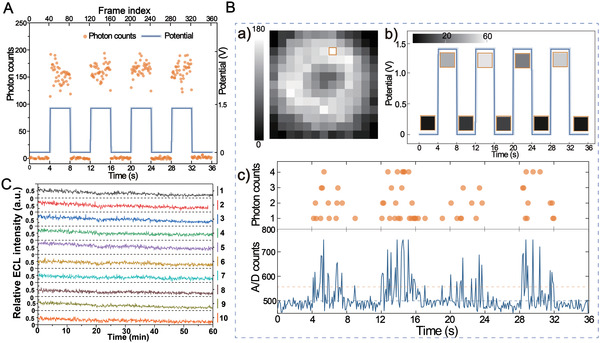
ECL visual details of nanoemitters at single‐molecule level. A) Synchronization signals for voltage and per frame photon counts of individual RuMOF emitter. B) the photon counting image of individual emitter from 360 frames a), and synchronization signals for voltage and 40 frame photon counting images b), and for per frame photon counts and the analogue‐to‐digital (A/D) counts c) of the single image pixel marked in a), the light blue dashed indicates the threshold to identify single photon and the yellow dashed indicates single‐photon intensity. Exposure time is 0.1 s and electron‐multiplying gain is 300 for the synchronization signals. C) Corresponding ECL trajectory of the same set of 10 ECL nanoemitters marked in the (Figure S10A, Supporting Information).

The amount of Ru(bpy)_3_^2+^ in one nanoemitter was determined through a single‐molecule perspective based on single Ru(bpy)_3_^2+^ scalar, showing 600 ± 37 Ru(bpy)_3_^2+^ molecules in single RuMOFs (more details in the Supporting Methods and Figure S9, Supporting Information). Despite that the amount is much lower than in the previously reported RuDSN (≈3.6 ×10^4^),^[^ ^7^ ^]^ the statistical analysis of the ECL signal‐to‐noise (S/N) exhibits that the value from RuMOF is fourfold larger than that from RuDSN (Figure 1E,F). Thus, the confined‐environment endows RuMOFs with more favorable ECL characteristics. In order to deeply understand the ECL emission from single nanoemitter, the photon counts collected on single emitter are measured and shown in **Figure** **2**A; and Movie S1 (Supporting Information). Synchronously variable behavior with relatively stable photon output corresponds to the voltage region (invalid voltage: 0 V or valid voltage: 1.35 V), respectively. This behavior reveals that the ECL optical events generated within a single RuMOF emitter unit are effectively controlled by voltage, having about 1600 photons generated per second from one emitter. To discriminate single photon spikes from offset noises (Figure 2B more details in the Supporting Methods), we further attempted to collect a single‐molecule ECL event on single imaging pixel (Figure 2B, a). Figure 2B, c indicates that the ECL optical signals were output synchronously with the voltage. Appreciable photon counts are collected that could be clearly resolved from offset noises, and the difference of photon counts could be visually differentiated by signal superposition from photon imaging mode (Figure 2B, b). It is pointed out that, compared with the surface‐modified/dye‐doped‐nanomaterials (i.e., RuDSN/AuNPs), the unique multipore confined‐space increases the reaction sites of Ru(bpy)_3_^2+^ with TPrA to facilitate the electron/proton transfer, resulting in increased photon generation efficiency. Additionally, this ECL nanoemitters with excellent high confined‐specific surface area and porosity can greatly improve the accumulation of intermediate radicals, which can prolong the ECL emission.^[^ ^25^ ^]^ After all, the electron/proton transfer rate and intermediates concentration play crucial roles in the ECL responses leading to single ECL events, which is proportional to the amount of captured ECL light.^[^ ^26^ ^]^ The excellent ECL intensity and stability of emitters are the cornerstone for successful imaging single bio‐sample on living cells with single‐molecule ECL approach. To the best of our knowledge, this is a pioneering exploration to directly collect single ECL events with single‐nanoemitter ECL reactions rather than in an aqueous solution with highly concentrated electroactive substances.^[^ ^5^ ^]^ Noteworthy, in the ECL application, a major drawback is the significant decrease in the ECL intensity over time that limits the application of ECL‐based microscopy for the dynamic study.^[^ ^27^ ^]^ This important decrease occurs very rapidly after a few seconds and this issue is usually underestimated or not mentioned in the ECL field. After the collection of ECL images from the nanoemitters under continuous applied voltage (Figure S10A, Supporting Information), 10 spots are randomly selected to analyze their respective ECL trajectories (Figure 2C; and Movie S2, Supporting Information). Within 60 min of imaging acquisition, the ECL intensity of all these spots remain stable with only a moderate decrease. Almost no strong ECL blinking is observed, which minimizes the possibility for the reduction of ECL efficiency (Figure S10B, Supporting Information).^[^ ^28^ ^]^

**Figure 3:**
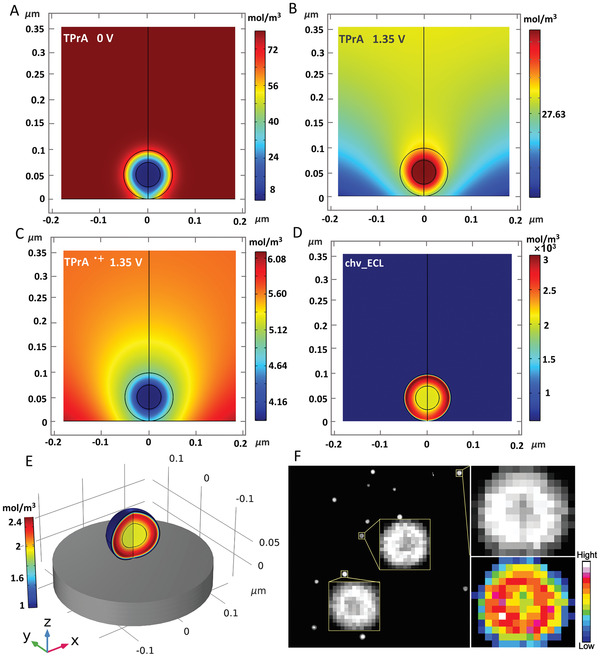
Dynamic analysis of microscopic imaging mechanism. A) Simulated concentration distribution of co‐reactant TPrA when it is initially added to the system before oxidation. B) The simulated kinetic concentration distribution of coreactant TPrA and C) TPrA^●+^ after oxidation. D) Side view of simulated ECL emission (i.e., the concentration distribution of Ru(bpy)_3_^2+⁎^), chv stands for the photon yield. E) The simulated 3D distribution pattern of Ru(bpy)_3_^2+⁎^. F) Single‐molecule images of single emitter, and the 2D spatial distribution ECL intensity is obtained by MATLAB analysis.

To better understand the nanoconfinement‐controlled imaging mechanism in RuMOFs, finite element simulation is performed to characterize the concentration distributions of TPrA radicals, and ECL emission of RuMOFs (details in Supporting Methods). At the potential of 1.35 V, the coreactant TPrA is first oxidized and then triggers Ru(bpy)_3_^2+^ to generate ECL emission (more details of reaction mechanisms in the Supporting Methods).^[^ ^7^, ^29^, ^30^ ^]^ Dynamic simulation shows that TPrA around and inside the RuMOFs is consumed (**Figure** **3**B), forming a highly local concentration of TPrA radicals (Figure 3C). We believe the enrichment of highly local concentration of reaction intermediates (TPrA radicals, Ru(bpy)_3_^+^) in nanochannel is the most powerful piece of the puzzle to adequately explain the nanoconfinement effect in enhancing ECL emission. Without the nanoporous structure, the TPrA radicals could move away from the electrode surface and diffuse into the bulk solution before randomly combining with the free Ru(bpy)_3_^2+^ to form Ru(bpy)_3_^2+⁎^.

With the nanoporous structure, the generated TPrA radicals are temporarily trapped in the nanoscale confined space. The continuously generation of highly local concentration of TPrA radicals can directly combine with Ru(bpy)_3_^2+^, increasing the generation rate of Ru(bpy)_3_^2+⁎^ and reducing energy loss due to the shorter electron conversion path. Thus, enhanced ECL is obtained, as evidenced by the ECL emission simulation (Figure 3D). Figure 3E shows the 3D simulation of Ru(bpy)_3_^2+⁎^ concentration distribution inside RuMOFs, which also represents the position of Ru(bpy)_3_^2+⁎^ and the 3D pattern of ECL emission (Figure S12, Supporting Information). Clearly, the outer load of Ru(bpy)_3_^2+⁎^ in RuMOFs is higher than the inner load, resulting in a difference in the intensity of ECL emission. This point is verified by single‐molecule imaging analysis of single RuMOF (Figure 3F), which is consistent with the 2D spatial distribution ECL intensity analysis.

To ensure that each ECL imaging spot corresponds to a single protein molecule, we used Cy5‐labeled Sgc8 aptamers as single‐molecule signal probes (Figure S13A, Supporting Information). The images indicate that most of the signal spots show photobleaching and photoblinking features with only a single‐step intensity trajectory, which suggests that each signal spot only corresponds to a signal probe (Figure S13B,C, Supporting Information). Under the same monitoring conditions, the spots number of ECL nanoemitters is basically the same as that of Cy5‐aptamer probes in the same size visible region (Figure S13E,F, Supporting Information). These results indirectly indicate that a single ECL spot represents a protein molecule.

Subsequently, we also investigated the visual monitoring of different quantities of targets. More target proteins (≈0.025≈25 ng) were modified at Ab1/ITO electrode, and the total number of ECL spots is related to the amount of target proteins. That means, more ECL spots are obtained in different amounts increase of targets (Figure S13G,H, Supporting Information). This result demonstrates that the proposed method can be used for accurate and effective visualization to monitor different amounts of targets, and can also be further applied as a sensitive single‐molecule detection or quantification strategy.

**Figure 4:**
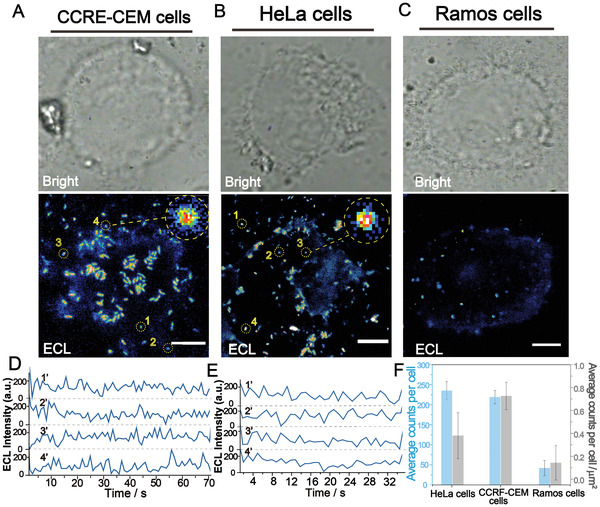
The verification of the accuracy and effectiveness of ECL nanoemitters approach monitoring single proteins on living cells. A) Bright‐field and ECL image of CCRF‐CEM cells. Scale bars, 3.5 µm. B) Bright‐field and ECL image of HeLa cells. Scale bars, 6 µm. C) Bright‐field and ECL images of Ramos cells. Scale bars, 5 µm. D) ECL trajectories of single‐molecule ECL signal spots marked in ECL image of A). E) ECL trajectories of single‐molecule ECL signal spots marked in ECL image of B). F) The average number of ECL spots on single cell (blue) and per square micrometer in per cell (gray) including HeLa cells, CCRE‐CEM cells and Ramos cells. The error bars were obtained by analysis of 20 cells.

Aiming to the ECL visualization of biomolecules on living cells by the present strategy, we selected the PTK7 over‐expressing CCRF‐CEM cell lines (suspension cells)^[^ ^31^ ^]^ and HeLa cells lines (adherent cells) as practical application models to assess the effective biological cognizance of the ECL nanoemitters. After labeling the ECL nanoemitters on cell surface through the specific recognition of Sgc8 aptamers and PTK7 protein (more details in the Supporting Information), ECL and PL images were recorded in situ (**Figure** **4**A,B; and Figures S14 and S15, Movies S3 and S4, Supporting Information).

The PL signal reflects the general location of ECL nanoemitters binding on cell membrane and displays a relatively intense signal from the entire cell, which demonstrate the immune recognition of ECL nanoemitters with the target proteins. Inevitably, strong background and autofluorescence (Figure S15, PL image and PL peak intensity, Supporting Information) are present.

By contrast with PL images, the ECL images of the same cell are clearer with a quasizero background luminescence (Figure 4 ECL images; and Figure S15 ECL peak intensity, Supporting Information). Consistent with the results obtained from imaging information, the emission peak profile of cell ECL images recorded in this work is significantly different from that of cell PL images (Figure S15A,D, Supporting Information). PL profile shows strong peak intensity across the whole cell, while the ECL profile signal peak shows only at the valid signal spots (Figure 4 ECL images and D, E the single‐spot representative trajectories). The size of some ECL imaging spots is measured to be 300 nm, which is similar to ECL spot observed from single protein ECL imaging (Figure S16, Supporting Information). Therefore, these ECL images should provide the information about individual proteins. Thus, the optical properties of such ECL points were applied as parameter thresholds to calculate the number of PTK7 proteins imaging in different cells (Figure 4F). Further, the PTK7 low‐expressing Ramos cells were tested with the same treatment conditions as the CCRF‐CEM cells (Figure 4C) and some sparse ECL spots are observed, which is only one‐fifth the content of CCRF‐CEM cells, in terms of individual cells (Figure 4F). These results are consistent with the visualized experiment and theoretical perspective of antigens on the cellular membrane, indicating the validity and accuracy of the proposed single‐molecule ECL strategy for in situ monitoring the occurrence and visualized quantification of target biomolecules on living cells. Gratifyingly, the present strategy successfully overcomes the limitations of external light source and background interference on the analysis and monitoring of single biomolecules on living cell surfaces.

Even for the 3D reconstructed image (Z‐Stack image) of ECL nanoemitters distribution on the surface of HeLa cell, there is no interference problems caused by spatial accumulation of background signals during the PL imaging process (Figure S17A,D, Supporting Information). Also, as the ECL imaging region extends from the electrode surface to the *z*‐axis direction, the recorded ECL emission gradually becomes blurred until it disappears, which is consistent with the distance dependence of the ECL emission on the electrode surface (Figure S17C, Supporting Information).^[^ ^30^, ^32^ ^]^

**Figure 5:**
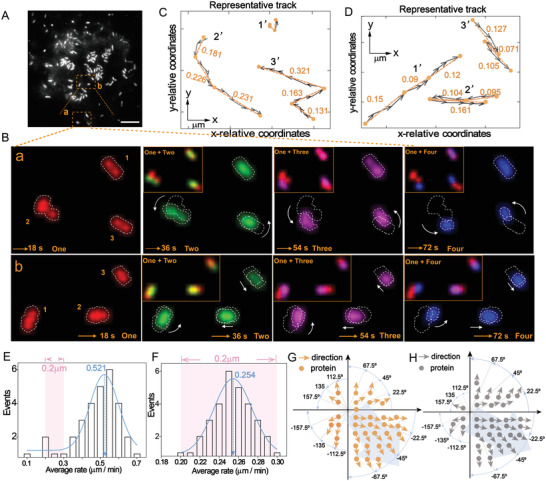
Real‐time ECL dynamic monitoring of membrane proteins. A) ECL image of single living cell for 72 frames. Scale bars is 3.5 µm. B) Dynamic monitoring of PTK7 proteins at different moments (18, 36, 48, and 72 s) for the selected a and b regions in A), the illustration shows an overlay of the current moment and the 18 s, and on the far right shows an overlay of the imaging at the four times and magnified images of representative movement proteins. C,D) Movement direction and relative position of protein from A) at different times in region a and b, respectively. E,F) Statistical analysis of the protein velocity of cellular peripheral region and medial region on single cell, respectively. The velocities were integrated in units of 0.05 and 0.01, respectively. G,H) Statistical analysis of the protein movement direction of cellular peripheral region and medial region on single cell, respectively. The data were analyzed with Image J and MATLAB.

Proteins in living cells are highly dynamic and generally do not remain at a certain site for a long time. Understanding their dynamic events can provide special information for deciphering the mechanisms about their contribution to cellular function.^[^ ^1^ ^]^ Highly stable emitter without the optical bleaching is the key to achieve high‐quality tracking, especially to visually monitor the dynamic process of membrane molecules. In view of the excellent ECL imaging characteristics of RuMOFs at the single molecule level, we further tracked the dynamic bio‐information of membrane proteins and recorded the movement status of different PTK7 individuals. The clear movements of individual proteins at the cellular membrane are clearly observed (**Figure** **5**A), which exhibits a high heterogeneity in the movement direction and velocity. For example, in the tracking of cell peripheral proteins (Figure 5A, region a), proteins at the sites of 2 and 3 tend to migrate unidirectionally with an average velocity of 0.530 and 0.494 µm min^−1^, respectively, while, protein at the site 1 barely moves (Figure 5B, a). Interestingly, for the proteins near the cell center (Figure 5A, region b), they have a relatively slow velocity (0.298, 0.300, and 0.245 µm min^−1^ at the sites 1, 2, and 3, Figure 5B, b). The proteins at the sites of 2 and 3 have the status of back‐and‐forth moving (Figure 5D). Indeed, statistical analysis of individuals dynamic properties in different regions (Figure S18A, Supporting Information) more intuitively reveals the differences in the velocity (Figure 5E,F) and trajectory direction (Figure 5G,H) of PTK7 individuals in the medial and peripheral regions of CCRE cells. The mainstream velocity of peripheral proteins (0.521 µm min^−1^) is obviously higher than that of the medial region (0.254 µm min^−1^), and the velocity range of different PTK7 individuals (0.1–0.7 µm min^−1^) is also wider than that of the medial region (0.2–0.3 µm min^−1^).

For the movement direction, the mainstream movement direction (from −90° to 0°) of protein populations in the two regions was consistent (Figure 5G,H). However, some proteins (up to 20%) completely deviated from the population, which might be potentially important information missed by the integrated or nonpremium single‐molecule measurement. The close association between the distribution and function of biomolecules may explain the dynamic heterogeneity of PTK7 proteins in the cytomembrane. As a signal transduction platform, membrane microdomains can make membrane proteins contact with related molecules, such as kinases, receptors, and signaling proteins. Consequently, proteins have corresponding reactivity and efficiency in the microdomain, and finally promote their functional or kinetic reactions.^[^ ^2^, ^33^ ^]^ Thus, membrane proteins can be compartmentalized into specific membrane regions where transverse organization, local composition, and dynamics differ in some way from average membrane properties. PTK7 proteins are involved in many physiological activities, such as migration, cell polarity, tissue regeneration, motility, and so on, but not all of these activities generate in the same membrane region.^[^ ^2^ ^]^ In this study, the peripheral PTK7 proteins exhibit more significant activity (relatively high movement velocity) and inter‐individual heterogeneity (wide velocity range) than the medial proteins. Thus, as high‐activity leader proteins on single cell, this will help PTK7 proteins to have a higher stress efficiency when it exerts its physiological role, and timely feedback cell to seek benefits and avoid harms. Likewise, similar results were obtained in the analysis of HeLa cells (Figure S19, Supporting Information).

The above findings can provide a reference for revealing the inter‐individual organization rules of membrane molecules and deepening the detailed cognition of individual biomolecules properties. As compared with the fluorescence observation, near‐zero ECL background surrounds the target protein with the ECL emitter, permitting a better contrast for the measurement of protein movement. Accordingly, a more accurate result could be obtained from the dynamic observation of ECL emitters in the continuous images.

## 3 Conclusion

In present study, we have designed a novel Ru(bpy)_3_^2+^‐embedded porous nanocomposite (MOF) as ECL nanoemitter to improve the accuracy and sensitivity, which is capable of monitoring biomolecules at single molecule level. Benefiting from the nanoconfinement effect, emitters are endowed with excellent ECL properties, enriching our visual cognition of discrete ECL events based on single nanoemitter ECL reactions, and improving the ECL intensity with long‐term stability. The developed nanoemitters and the associated single‐molecule ECL approach with almost zero‐background interference truly overcome this unavoidably limitation in current optical imaging for individual biomolecules. As a result, our single‐molecule ECL imaging system achieves dynamic mapping of biomolecules at the single molecule level and can perform real‐time ECL monitoring of single biomolecule on living cells surface, and effectively differentiating the heterogeneity of velocity and movement direction among protein individuals in different regions. This work constitutes a valuable contribution to steer meaningful exploration in this direction of ECL imaging and gain insightful visible information regarding cells or small biomolecules.

## 4 Experimental Section

Supporting Materials and Methods, Supporting Data (Figures S1–S20), Supporting References and Movies S1–S4 are provided in the Supporting Information).

## Conflict of Interest

The authors declare no conflict of interest.

## Supporting information

Supporting Information

Supplemental Movie 1

Supplemental Movie 2

Supplemental Movie 3

Supplemental Movie 4

## Data Availability

The data that support the findings of this study are available from the corresponding author upon reasonable request.
