# Supplementary material for: High Electrochemiluminescence from Ru(bpy)3 2+ Embedded Metal–Organic Frameworks to Visualize Single Molecule Movement at the Cellular Membrane
Source: Adv Sci (Weinh). 2022 Nov 3;9(35):2204715. doi: 10.1002/advs.202204715 (PMC9762315; doi:10.1002/advs.202204715)
Supplement: Supplementary file 1 — Supporting Information [file ADVS-9-2204715-s003.pdf]

ADVANCED SCIENCE
Open Access

Supporting Information

for *Adv. Sci.*, DOI 10.1002/advs.202204715

High Electrochemiluminescence from $Ru(bpy)_3^{2+}$ Embedded Metal–Organic Frameworks to Visualize Single Molecule Movement at the Cellular Membrane

Binxiao Li, Xuedong Huang, Yanwei Lu, Zihui Fan, Bin Li, Dechen Jiang, Neso Sojic* and Baohong Liu**

Supporting Information
©Wiley-VCH 2021
69451 Weinheim, Germany

High Electrochemiluminescence from $Ru(bpy)_3^{2+}$ embedded Metal–organic Frameworks to Visualize Single Molecule Movement at the Cellular Membrane

Binxiao Li,[a] Xuedong Huang, [a] Yanwei Lu, [a] Zihui Fan, [a] Bin Li, [a] Dechen Jiang,*[b] Neso Sojic*[c] and Baohong Liu*[a]

[a] Dr. B. Li, X. Huang, Y. Lu, Z. Fan, B. Li, and Prof. B. Liu
Department of Chemistry, Shanghai Stomatological Hospital, State Key Laboratory of Molecular Engineering of Polymers
Fudan University, Shanghai 200433 (China)
E-mail: bhliu@fudan.edu.cn

[b] Prof. D. Jiang
State Key Laboratory of Analytical Chemistry for Life and School of Chemistry and Chemical Engineering
Nanjing University, Nanjing, Jiangsu, 210093 (China)
E-mail: dechenjiang@nju.edu.cn

[c] Prof. N. Sojic
Bordeaux INP, institute of Molecular Science (ISM), and CNRS UMR 5255, University of Bordeaux, 33607 Pessac (France)
E-mail: neso.sojic@enscbp.fr

Table of Contents

Supplementary Materials and Methods:
Materials
Apparatus and characterization
Synthesis of nanoconfined-RuMOFs nanoemitters
Preparation of nanoconfined-ECL nanoemitters
Preparation of aminated ITO
Fabrication of the ECL detection chip
Construction process of ECL biosensor
Cell culture and seeding
Measure of the number of $Ru(bpy)_3^{2+}$ per RuMOFs
Imaging instrumentation
Calculation of photon counts
COMSOL simulation for ECL pattern analysis

Supplementary Figures:
Figure S1. Setup of the electrochemical (ECL) chip.
Figure S2. DLS verification of the synthesized RuMOFs.
Figure S3. TEM energy mapping of ZrMOF NPs and RuMOF nanoemitters.
Figure S4. Zeta potential of the ZrMOF NPs and RuMOFs.
Figure S5. Photographs of ZrMOF NPs and RuMOF nanoemitters in water.
Figure S6. Stability verification of the ZrMOF NPs and RuMOFs.
Figure S7. The peak intensity analysis of ECL signal spots from marked region.
Figure S8. ECL intensity distribution of ECL nanoemitters. At least 600 ECL nanoemitters were analyzed.
Figure S9. Measure of the Number of $Ru(bpy)_3^{2+}$ molecules per RuMOF.
Figure S10. ECL images of ECL nanoemitters and the representative ECL-time trajectory obtained from single ECL nanoemitter.
Figure S11. Geometry modeling of RuMOF in COMSOL software.
Figure S12. Simulated concentration distribution of $Ru(bpy)_3^{2+*}$ and 3D distribution pattern of ECL emission.
Figure S13. The verification of the accuracy and effectiveness of ECL nanoemitters approach monitoring single proteins..
Figure S14. Characterization of the bio-application performance of ECL nanoemitters on living cells.

Figure S15. Characterization of the optical performance of ECL nanoemitters on living cells.
Figure S16. Validation of single protein signals on the surface of living cells.
Figure S17. Characterization of the imaging performance of ECL nanoemitters on living cells..
Figure S18. Statistical analysis of proteins in different regions on single cell and the temporal evolution record of protein individuals,
Figure S19. Dynamic analysis of protein individuals in different regions of HeLa cells.
Table S1. Reactions involving the ECL process.
Table S2. Simulation parameters.
References

Experimental Procedures

Supplementary Materials and Methods

Materials

2,2′-bipyridine-5,5′-dicarboxylic acid (BPDC), Zirconium chloride ($ZrCl_4$), tris(2,2′-bipyridine) ruthenium (II) hexafluorophosphate-triethy lamine, tripropylamine (TPA), (3-Aminopropyl) triethoxysilane (APTES), glutaraldehyde (GLD), poly-L-lysine (PLL), bovine serum albumin (BSA, 98%) were obtained from Sigma-Aldrich (St Louis, USA). Dimethylformamide (DMF), ethanol, hydrogen peroxide (H_2O_2, 30%), acetic acid, acetone and ammonia solution ($NH_3 \cdot H_2O$, ~ 30%) were ordered from Sinopharm Chemical Reagent Co., Ltd. (Shanghai, China). Fetal bovine serum (FBS), RPMI 1640 cell culture medium, trypsin, and phosphate-buffered saline (PBS) were obtained from Gibco (USA). Human PTK7 recombinant protein and its mouse monoclonal antibodies (capture antibodies Ab_1) were ordered from Abnova (Taipei, Taiwan). Human acute lymphoblastic leukemia (CCRF-CEM) cells were obtained from Cell Bank of the Committee on Type Culture Collection of the Chinese Academy of Sciences. Human Burkitt's lymphoma (Ramos) cells and human cervical carcinoma (HeLa) cells were purchased from the Cancer Institute & Hospital (Chinese Academy of Medical Sciences). All reagents (analytical regent) were used with no further purification. The water used was purified on a Milli-Q Biocell Purification System (Biller, MA, USA). These DNA sequences used in this study, all HPLC-purified and lyophilized, were synthesized by Sangon Biotech. Co., Ltd. (Shanghai, China).
The sequences are as follows:
Phosphate-T10-Sgc8:

5'-$H_2PO^-_3$-TTT TTT TTT TAT CTA ACT GCT GCG CCG CCG GGA AAA TAC TGT ACG GTT AGA -3'

Phosphate-T10-Sgc8-Cy5:

5'-TTT TTT TTT TAT CTA ACT GCT GCG CCG CCG GGA AAA TAC TGT ACG GTT AGA-Cy5-3'

Apparatus and characterization

An Agilent HP8453 UV-vis spectrophotometer was used to record UV-vis absorption spectra. The fluorescence measurements were performed using a quartz cuvette and an F-4600 fluorescence spectrometer (Japan). The shape and size of Uio-67 were characterized with a JEOL JEM-2011 transmission electron microscope (TEM). A Zetasizer (Nano-Z, Malvern, UK) was used as Zeta potential and grading analysis. The single molecule and ECL imaging experiments were achieved with a home-built imaging system by Professor JW Liu.

Synthesis of nanoconfined-RuMOFs nanoemitters

The method of synthesising RuMOFs has been improved refer to previous works.[1,2] As the precursor solution of organic ligand, 45 mg of BPDC was dispersed into 20 mL of DMF contained 360 μL of triethylamine. As the white metal precursor solution, 45 mg of $ZrCl_4$ was dispersed into 16 mL of DMF with ultrasonic. Then, the two solutions were mixed with 2.25 mL of acetic acid, and the mixture was transferred into a Teflon sealed autoclave to obtain a high-pressure reaction and sat at 90 °C for 24 h. After the solvothermal reaction, the products were centrifuged at 6500 rpm for 12 min, then further washed with DMF, methanol and ethanol respectively. Ultimately, the white precipitate was obtained. The product was dried and used for further experiments or dispersed at ethanol with a concentration of 2 mg/mL. Whereafter, the tris-(2,2′-bipyridine) ruthenium (II) hexafluorophosphate ($Ru(bpy)_3(PF_6)_2$) was incorporated into the ZrMOF nanoparticles. Simply, 10 mL of $Ru(bpy)_3(PF_6)_2$ DMF solutions (2 mg/mL) was mixed with 10 mL of the UiO-67 dispersion to obtain orange yellow solution. The solution was then stirred at 95 °C for 15 h, followed by removal of the DMF solvent and centrifugation with DMF and ethanol. The obtained yellow solid was dried or dispersed in ethanol and store at 4 °C for further work.

Preparation of nanoconfined-ECL nanoemitters

The RuMOFs were first washed with ultrapure water twice (6500 rpm, 12 min) to remove ethanol and resuspended in DMF. The phosphate-terminal labeled aptamers (phosphate-T10-Sgc8 and phosphate-control) (100 μL, 10 μM) were added to freshly synthesized RuMOFs (2 mg/mL) and the mixture was incubated under gentle shaking for 5 h at room temperature. Then, free aptamers were removed by centrifugation and washing with water, the conjugates were redispersed in buffer/water. Finally, the ECL probe was obtained and stored at 4 °C until use.

Preparation of Aminated ITO

Firstly, the indium tin oxide (ITO) coated cover slips (0.13-0.17 mm thick, 20 × 20 mm, ~ 15-30 Ω, SPI Supplies, USA) were cleaned by sonication in acetone, ethanol, and ultra-pure water for 12 min respectively. Then, they were dried by N_2 stream and etched in vacuum plasma (PDC-002, Harrick Plasma Inc., U.S.) for 8 min. Whereafter, the ITO slides were dipped into the mixture solution of $H_2O_2/NH_3{\cdot}H_2O/H_2O$ (1:1:5, V/V) for 0.5 h to generate an active hydroxyl layer and washed twice times with ultrapure water. After drying by a N_2 stream, the ITO slides were immersed in 0.5% APTES (ethanol) for 0.5 h at room temperature, ultrasonized 30 s and rinsed twice quickly with ethanol, and heated at 80 °C for 30 min. Finally, after the washing with ultrapure water, the amine groups were successfully introduced on the ITO electrode.

Fabrication of the ECL detection chip

The simple and convenient detection chip was composed of an ITO electrode, a square PDMS and a carbon strip. Firstly, four holes were punched at the central part of a square PDMS by puncher as reservoirs. Then, the PDMS was etched in vacuum plasma for 10 min. Afterwards, the PDMS layer was attached to the modified ITO piece surface and pressed firmly. Then, with conductive silver paste (SPI, USA), a carbon strip was attached to one corner of the modified ITO. The prepared chips were stored for further use.

Construction process of ECL biosensor

A sandwich interface was constructed on the aminated ITO slice by stepwise modification. 30 μL of 5% GLD was added into the detection chip to activate the amine groups for 1 h at room temperature. Then, 30 μL of 0.1 μg/mL Ab_1 was introduced onto the GLD/ITO activated surface by covalent coupling. After washing gently with 0.01 M PBS, the detection chip was exposed to 30 μL of 1% BSA for 1h at 37 °C. Whereafter, different concentrations of PTK7 solution (30 μL) were added to this detection chip and left at 37 °C for 1h. 30 μL of ECL emitters were then dropped onto this detection chip and incubated at 37 °C for 0.5 h. Finally, the chips were washed thoroughly with PBS to remove free ECL probe or nonspecifically bounded conjugates.

Cell culture and seeding

CCRF-CEM cells, Ramos cells and Hela cells were cultured in RPMI 1640 medium, with 10% fetal bovine serum and 0.5 mg/mL penicillin-streptomycin at 37 °C under a 5% CO_2 atmosphere. For the suspension cells (CEM cells and Romas cells), the ITO electrodes were etched in vacuum plasma for 6 min and then immersed into 0.01% (w/v) PLL solution for a 10 min incubation. This step could can increase cell adhesion of the sensing interface.[3] The CEM cells and Romas cells in the exponential growth phase were centrifuged at 1000 rpm for 3 min and washed with PBS. The cell number was determined using a hemocytometer. Then, cells were incubated in cell culture medium containing the ECL probes (25 μg/mL), which were shaked gently for 45 min h at 37 °C to label the probes on cell surface through the specific recognition of Sgc8 aptamers and PTK7 protein on cell surface. The medium was then removed and the cells were washed gently with PBS. After that, the suspension cells were seeded on the pole of the PLL/ITO and incubated 0.5 h for ECL imaging.

For the adherent cells (Hela cells), the ITO electrodes were etched in vacuum plasma for 6 min. The Hela cells in the exponential growth phase were digested for 2 min by trypsin (0.1%, m/v), and then separated from the cell culture medium. The cells were gathered by the centrifuging at 800 rpm for 2 min, and then, washed with PBS. The cells were then resuspended in cell culture medium and seeded on the pole of the ITO. 10 h (37 °C, 5% CO_2) incubation was applied for 10 h to achieve the immobilization of cells. Then, the freshly culture solution containing the ECL probe (25 μg/mL) was added and incubated for 60 min at 37 °C. After washing cells, the single cell ECL imaging were performed.

Measure of the number of $Ru(bpy)_3^{2+}$ per RuMOFs

First, we collected the photoluminescence information of a single $Ru(bpy)_3^{2+}$ molecule, as shown in **Figure S9**. Then, 500 imaging signal points were selected for PL intensity analysis to obtain the average intensity ($\bar{p}$ =0.85) of a single $Ru(bpy)_3^{2+}$ (**Figure S9**). Under the same imaging and analysis conditions, the PL signals of 500 emitters were statistically analyzed to obtain their intensity information ($P = p \pm W$ =510 $\pm$31), as shown in **Figure S8**. After comparing the intensity with that of a single Ru molecule, the amount of Ru molecules contained in each RuMOF was obtained (Q_p =600 ± 37). W represents for half-peak width intensity value.

$$Q_p = \frac{P}{\bar{p}} = \frac{p \pm W}{\bar{p}} = 600 \pm 37$$

Imaging instrumentation and data analysis

The PL/ECL/optical imaging was operated on a homemade imaging system, which was composed of a wide-field epi-fluorescence microscope (Chiyoda, Tokyo, Japan), an ultrasensitive electron multiplying charge coupled device (EM-CCD) (Ixon DU888, Andor Technology Plc., U.K.) and a ECL detection chip, shown in **Figure S1**. The microscope was installed in a self-made dark box to avoid interference from outside light. A motorized microscope stage and oil immersion objective len (NA 1.45, 100×, Olympus, Japan) was equipped for the sample recording. ECL detection chip was used in a two-electrode configuration, in which the modified ITO electrode acted as working electrode, and a Pt wire as counter electrode. Moreover, this imaging system included a potentiostat from the voltage generator (DG 1021, Rigol, China). A switching of the potential between +1.35 V and 0 V was continuously employed on the work electrode to trigger the ECL reaction. TPrA (0.08 M) was chosen as an oxidative-reduction sacrificial coreactant. The EMCCD was triggered and synchronized by the potentiostat. All measurements were performed at room temperature. The data analysis method is implemented from the Professor W.E. Moerner lab to recognize single molecule spots in images with some modification.[3,4] In this work, the data were mainly analyzed with ImageJ and home-written MATLAB.

Calculation of photon counts

The A/D (analog-to-digital) counts of the camera is described below.

$$A/D\ Counts = \frac{N \times QE \times EM\ Gain}{A/D\ unit}$$

where N is the number of photons (photon counts) incident to the pixel, QE is the photoelectric conversion efficiency, electron-multiplying (EM) Gain is the electron multiplication coefficient of the camera. A/D unit is the analog-to-digital conversion coefficient that is 5.67 for the EMCCD. The EM Gain selected in the experiment is 300 and the QE is viewed as 1.

COMSOL simulation for ECL pattern analysis

The experimental configuration was modeled using the commercial finite element software (COMSOL). In this simulation, a 2D axisymmetric model was built to simulate the physical geometry in ECL experiments, as shown in **Figure S11**. A physical geometry consisting of porous nanoparticles, electrochemical reaction cell, supporting ITO electrode and electrolyte was constructed in the electrical analysis module of COMSOL software. The boundary and domain settings were shown in **Figure S11**. The charge transfer reaction occurred on the surface of the supporting electrode with potential of 1.35 V (domain 2). The homogeneous chemical reaction occurred in the electrolyte (domain 1). A porous nanoparticle of diameter 100 nm loaded with $Ru(bpy)_3^{2+}$ was located in the middle of the electrode. The kinetic and thermodynamic parameters were referred to previously reported works, except for the diffusion coefficient of TPrA and Ru-complex were adjusted accordingly.[5]

Table S1. Reactions involving the ECL process.

(1)	$TPAH^+ \leftrightarrow TPA + H^+$	$K_1 = \frac{k_1}{k_{-1}}$ (K1 forward rate,K-1 backward rate)
(b)	$BufH^+ \leftrightarrow Buf + H^+$	$K_b = \frac{k_b}{k_{-b}}$
(e)	$H_2O \leftrightarrow OH^- + H^+$	$K_e = \frac{k_e}{k_{-e}}$
(2)	$TPrA - e \xrightarrow{k_{S2}} TPrA^{\bullet+}$	
(3)	$TPrA^{\bullet+} \xrightarrow{k_3} TPA^{\bullet} + H^+$	$K_3 = \frac{k_3}{k_{-3}}$
(4')	$TPrA\bullet - e \xrightarrow{k_{S4'}} P1$	
(4)	$\vert{-}Ru^{2+} + TPrA^{\bullet} \xrightarrow{k_4} \vert{-}Ru^{+} + P1$	P1, the product of TPrA• oxidation
(5)	$\vert{-}Ru^{+} + TPrA^{\bullet+} \xrightarrow{k_5} \vert{-}Ru^{2+*} + TPrA$	
(6)	$\vert{-}Ru^{2+*} \xrightarrow{k_{des}} \vert{-}Ru^{2+} + hv$	

Table S2. Simulation parameters.

Compound	Total initial concentration	Diff.coeff. D (cm^2 /s)	Thermo.	Rate constants	
Buf/$BufH^+$	0.3	$5*10^{-6}$	$K_{buf} = 10^{-8}$	$k_b = 2*10^3$	$k_{-b} = 3*10^{10}$
H^+	10^{-8}	$9.3*10^{-5}$	$K_e = 10^{-14}$	$k_e = 2*10^{-3}$	$k_{-e} = 3*10^{10}$
TPrA/$TPrAH^+$	0.08	$5*10^{-7}$	$K_1 = 10^{-10.4}$	$k_1 = 8$	$k_{-1} = 3*10^{10}$
TPrA/$TPrA^{\bullet+}$			$E_0 = 0.88^b$	$k_{s2} = 10$	
$TPrA^{\bullet+}$		$5*10^{-7}$	$K_3 > 10^3$	k_3 adjusted	$k_{-3} = 0.07$
$TPrA^{\bullet}$		$5*10^{-7}$	$E^0 = -1.7^b$	$k_{s4'} = 10$	
Ru^{2+}	10^{-9}	$0 - 5*10^{-14}$		$k_4 > 3*10^5$	
Ru^+		$0 - 5*10^{-14}$		$k_5 > 3*10^5$	
Ru^{2+*}		$0 - 5*10^{-14}$		$k_{des} > 3*10^2$	

(Notes: all solution concentrations are in M, surface concentrations in mol cm^{-2}, for all reaction except i=4 and 5, K_i in M, k_i in s^{-1} and k_{-i} in $M^{-1}s^{-1}$, k_{s2} and $k_{s4'}$ in cm/s, $k_{4,5}$ in $M^{-1}s^{-1}$, k_{des} in s^{-1}.) In numerical simulation, charge transfer reaction and mass transport were considered in the electrochemical reaction. The mass transport was determined by the Nernst-Planck equation.[6]

$$\frac{\partial C_i}{\partial t} + \nabla J_i = R_i,$$

$$J_i = -D_i \nabla C_i - \frac{Z_i}{RT} D_i C_i \nabla \varphi.$$

The oxidation and reduction of (2)(4) follow the Butler-Volmer equation:

$$i = FAk^0[C_O(0,t)e^{\frac{-\alpha F \eta}{RT}} - C_R(0,t)e^{\frac{(1-\alpha)F\eta}{RT}}]$$

where the transfer coefficient α was taken as 0.5.
The effective transfer coefficient of porous materials was corrected by Bruggeman[7]:

$$D_{i,eff} = \epsilon_l^{1.5} D_i$$

where is the porosity, which is set to 0.9 in the center and 0.1 at the edge of the nanooporous MOF.

Results and Discussion

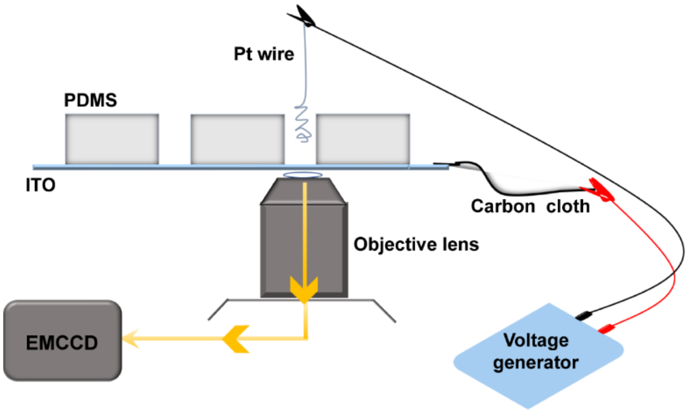

Figure S1. Setup of the electrochemical (ECL) chip.

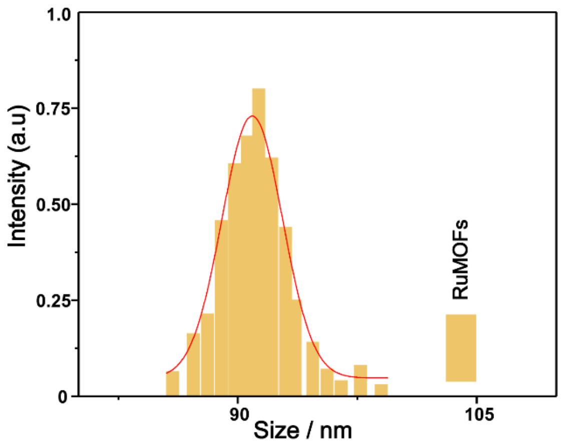

Figure S2. DLS verification of the synthesized RuMOFs.

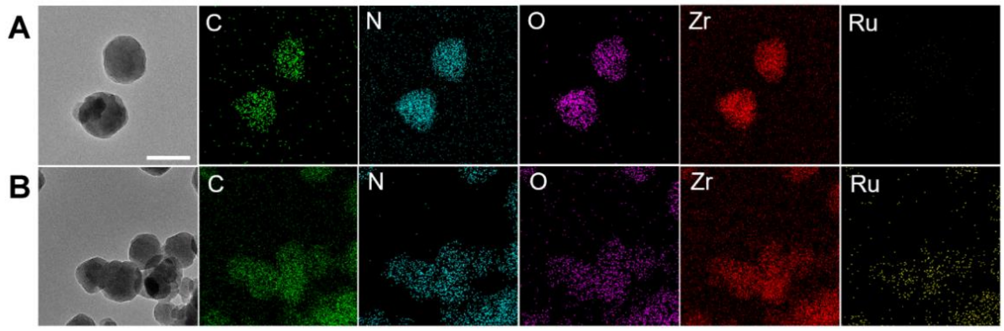

Figure S3. TEM energy mapping of ZrMOF NPs and RuMOF nanoemitters. (A) The ZrMOF NPs and (B) the @RuMOFs nanoemitters.

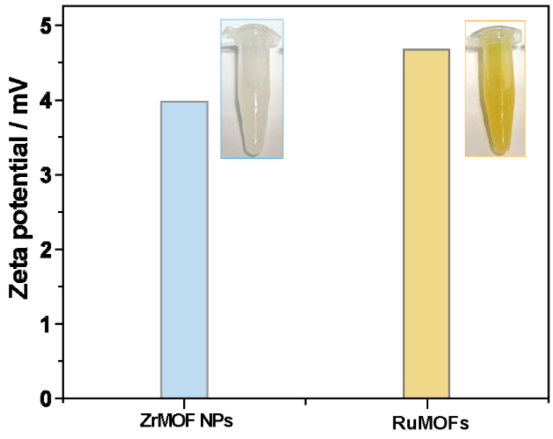

Figure S4. Zeta potential of the ZrMOF NPs and RuMOFs. The light blue represents ZrMOF NPs and the light yellow represents RuMOF nanoemitters.

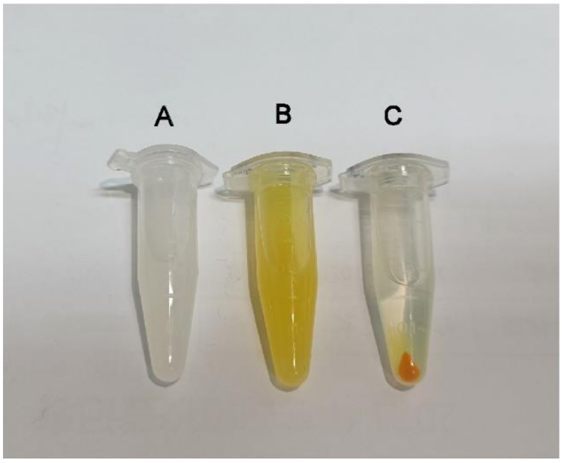

Figure S5. Photographs of ZrMOF NPs and RuMOF nanoemitters in water. (A) The ZrMOF NPs dispersion in water, and the RuMOFs nanoemitters before (B) and after centrifugation (C) in water.

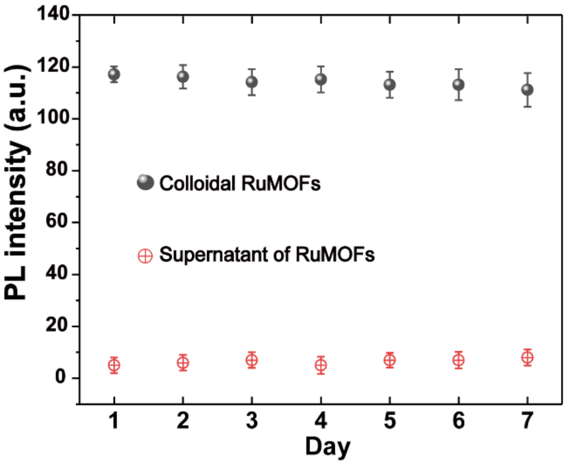

Figure S6. Stability verification of the ZrMOF NPs and RuMOFs. PL intensity of colloidal RuMOFs and supernatant of RuMOFs after centrifugation under different storage days.

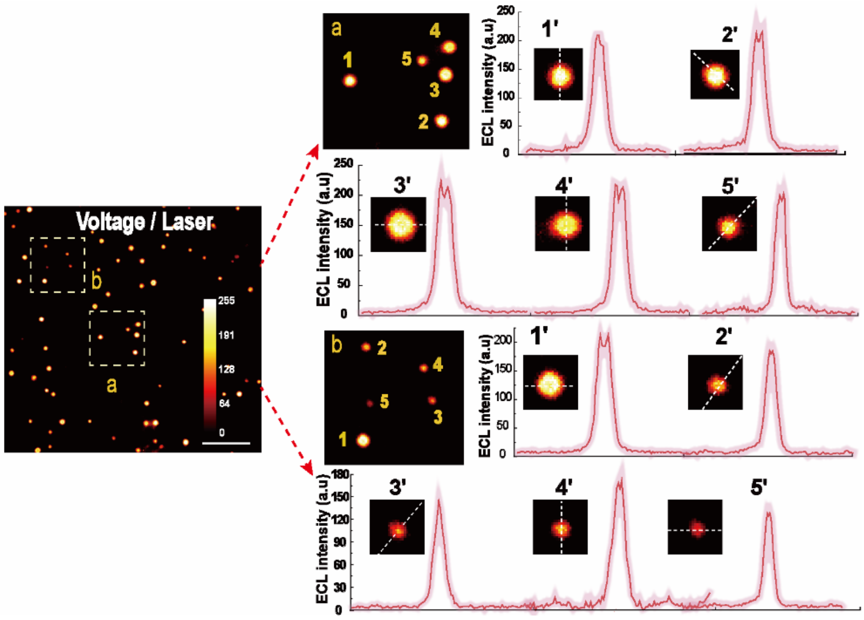

Figure S7. The peak intensity analysis of ECL signal spots from marked region. The selected area is the representative visible dark area.

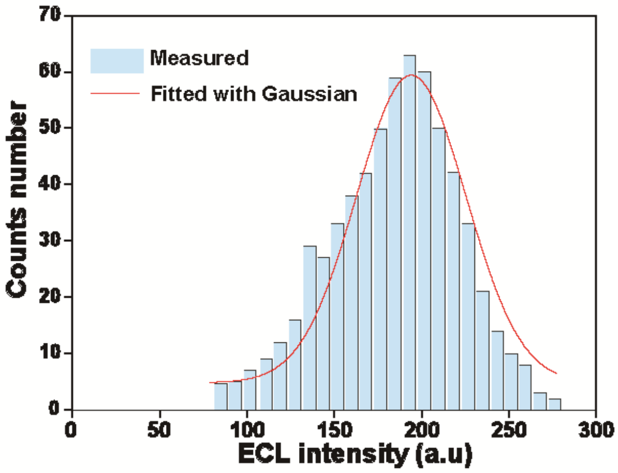

Figure S8. ECL intensity distribution of ECL nanoemitters. At least 600 ECL nanoemitters were analyzed.

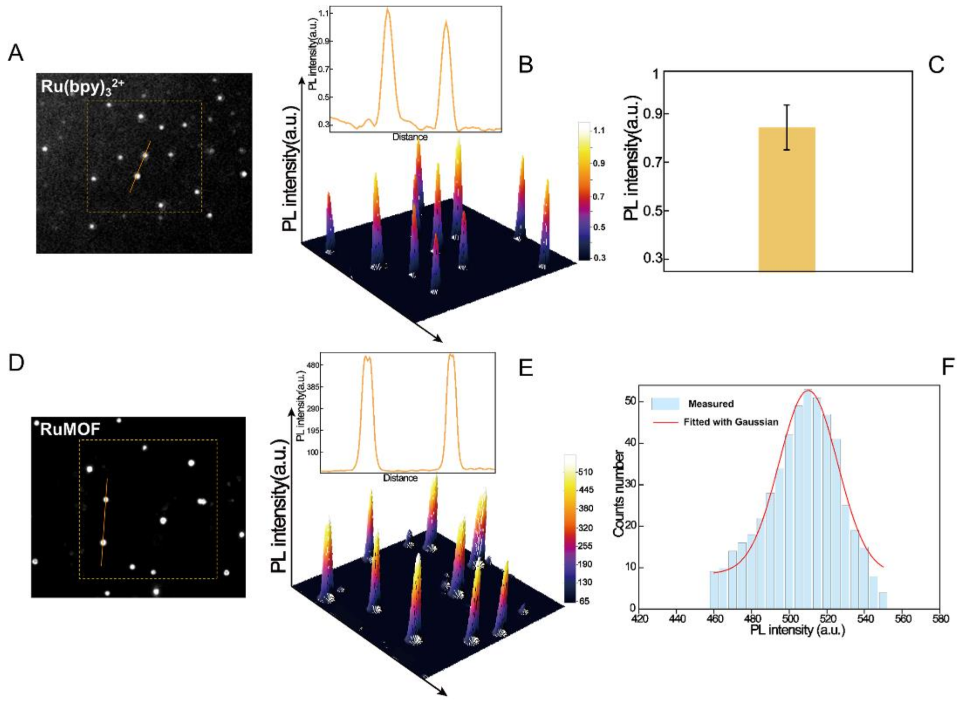

Figure S9. Measure of the Number of $Ru(bpy)_3^{2+}$ molecules per RuMOF. (A) single-molecule PL imaging of $Ru(bpy)_3^{2+}$ molecules. (B) Representative 3D intensities of $Ru(bpy)_3^{2+}$ from marked region in (A), the inset is the peak intensity of the PL signal points from two $Ru(bpy)_3^{2+}$ molecules along the yellow line in (A). (C) The average PL intensity of a single $Ru(bpy)_3^{2+}$. (D) RuMOFs single-molecule PL imaging. (E) Representative 3D intensities of RuMOF from marked region in (D), the inset is the peak intensity of the PL signal points from two RuMOF along the yellow line in (D). (F) The PL intensity statistical distribution of RuMOF nanoemitters. 500 nanoemitters were analyzed.

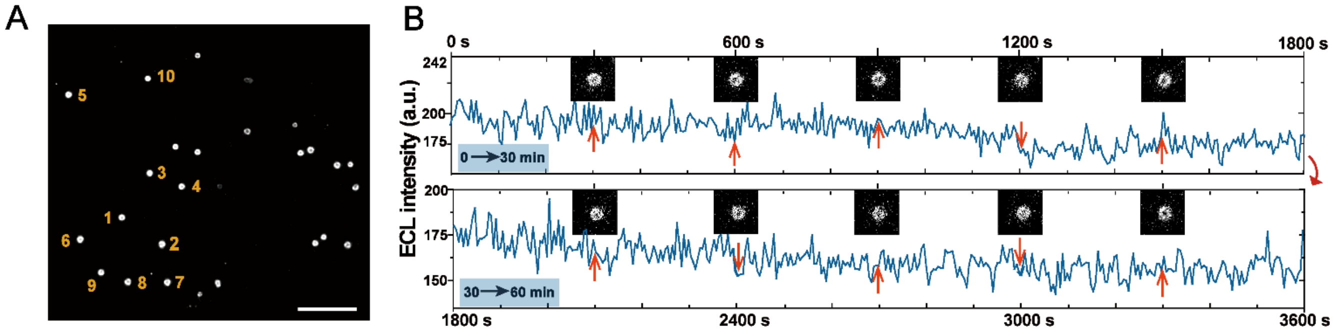

Figure S10. (A) ECL images of ECL nanoemitters recorded under the applied voltage. Scale bars (white), 3 μm. (B) The representative ECL-time trajectory obtained from single ECL nanoemitter after being captured by a single target protein, the insets show the ECL images at the trajectory pointed by the red arrow.

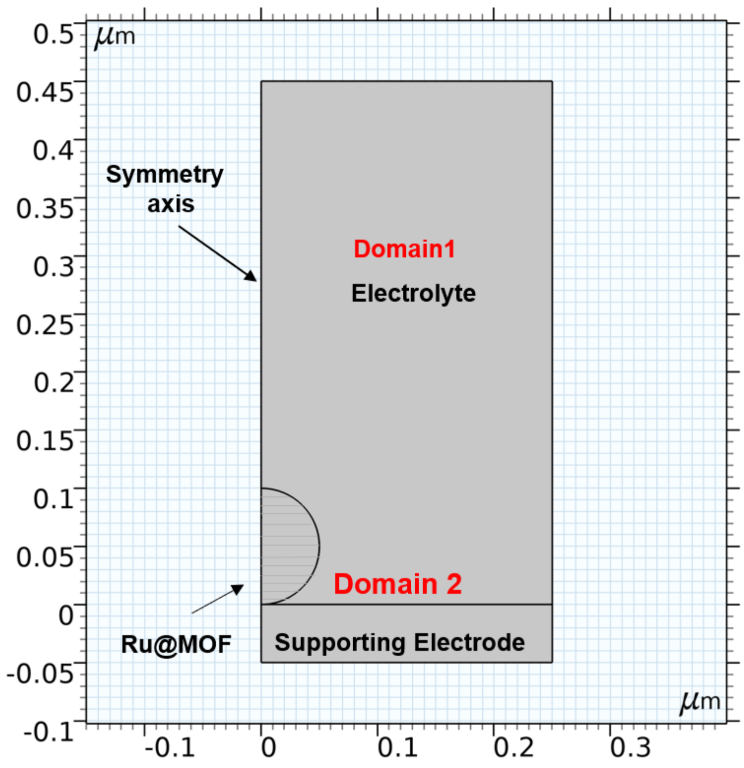

Figure S11. Geometry modeling of RuMOF in COMSOL software.

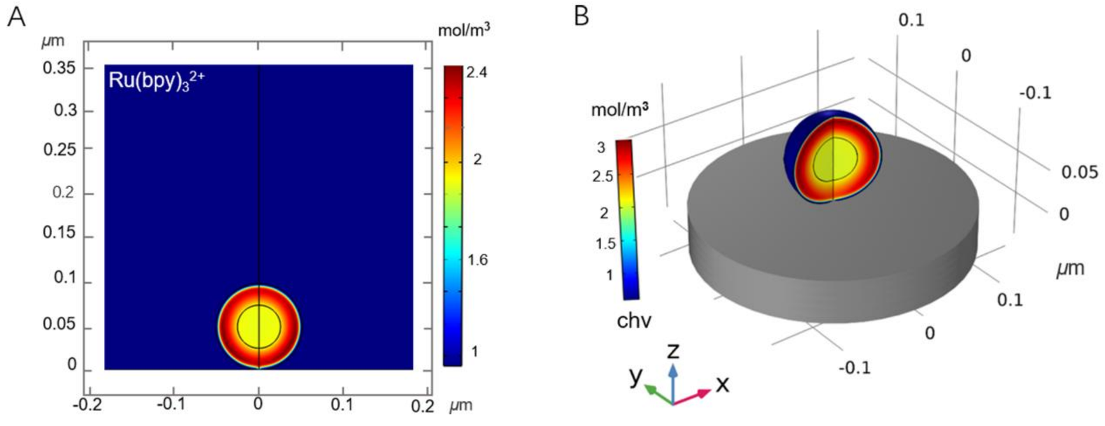

Figure S12. (A) Side view of simulated concentration distribution of $Ru(bpy)_3^{2+}$*. (B) The simulated 3D distribution pattern of ECL emission, chv stands for the photon yield.

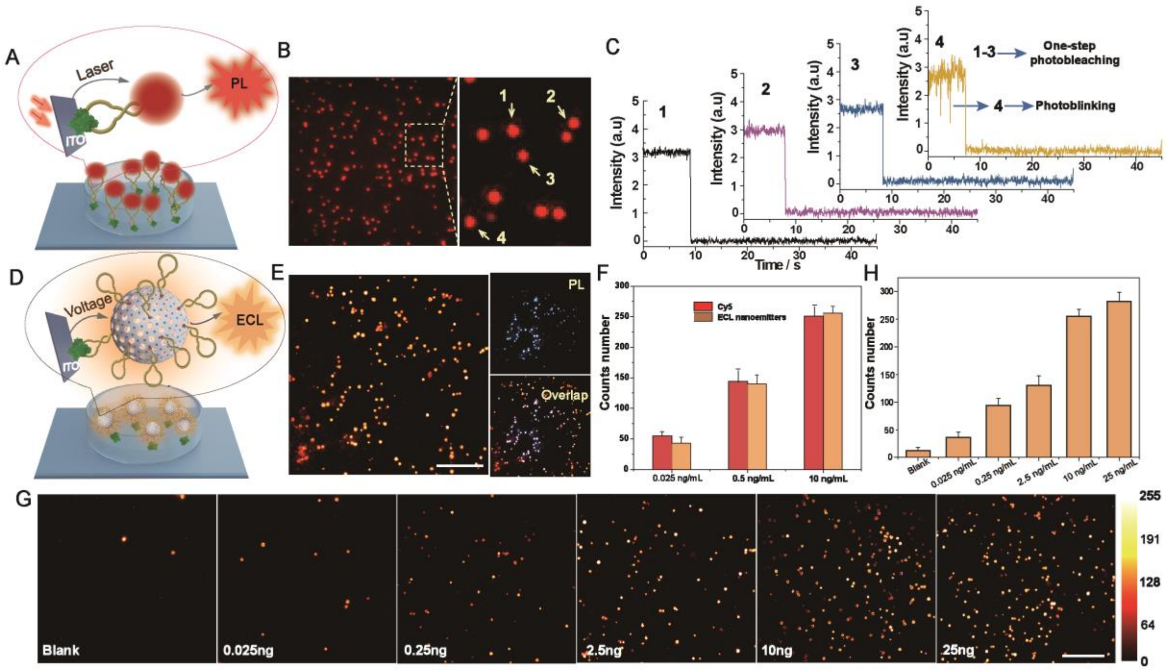

Figure S13. The verification of the accuracy and effectiveness of ECL nanoemitters approach monitoring single proteins. (A) Scheme of Cy5/aptamer labeled proteins. (B) Single molecule fluorescent images of Cy5 in the experiment (A). (C) Repre-sentative traces with one-step photobleaching and photoblinking (also photobleached in one step at the end) of Cy5 in the images of (B). D) Schematic of ECL nanoemitters labeled proteins. E) ECL images of ECL nanoemitters in the experiment (D). Scale bars (white), 5 μm. (G) ECL images of ECL nanoemitters with various PTK7 protein concentrations. (F) Counting of fluorescent spots and ECL spots at different concentrations of proteins (0.025,0.5 and 10 ng/mL). (H) The plot of the number of signal spots from ECL nanoemitters with various PTK7 protein concentrations in the range from 0 to 25 ng/mL. Scale bars (white), 5 μm.

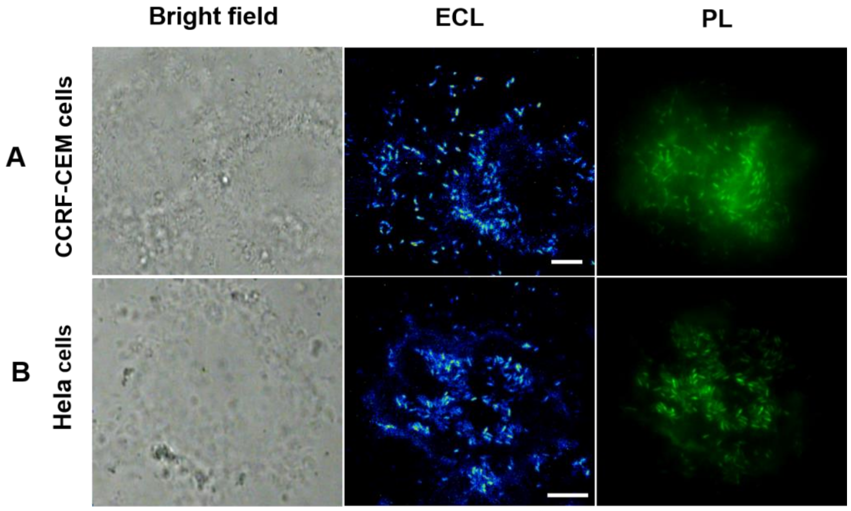

Figure S14. Characterization of the bio-application performance of ECL nanoemitters on living cells. Bright-field, ECL and PL images of PTK7 on the surface of the CCRF-CEM cells and the Hela cells. Scale bars, 3 μm and 5 μm respectively.

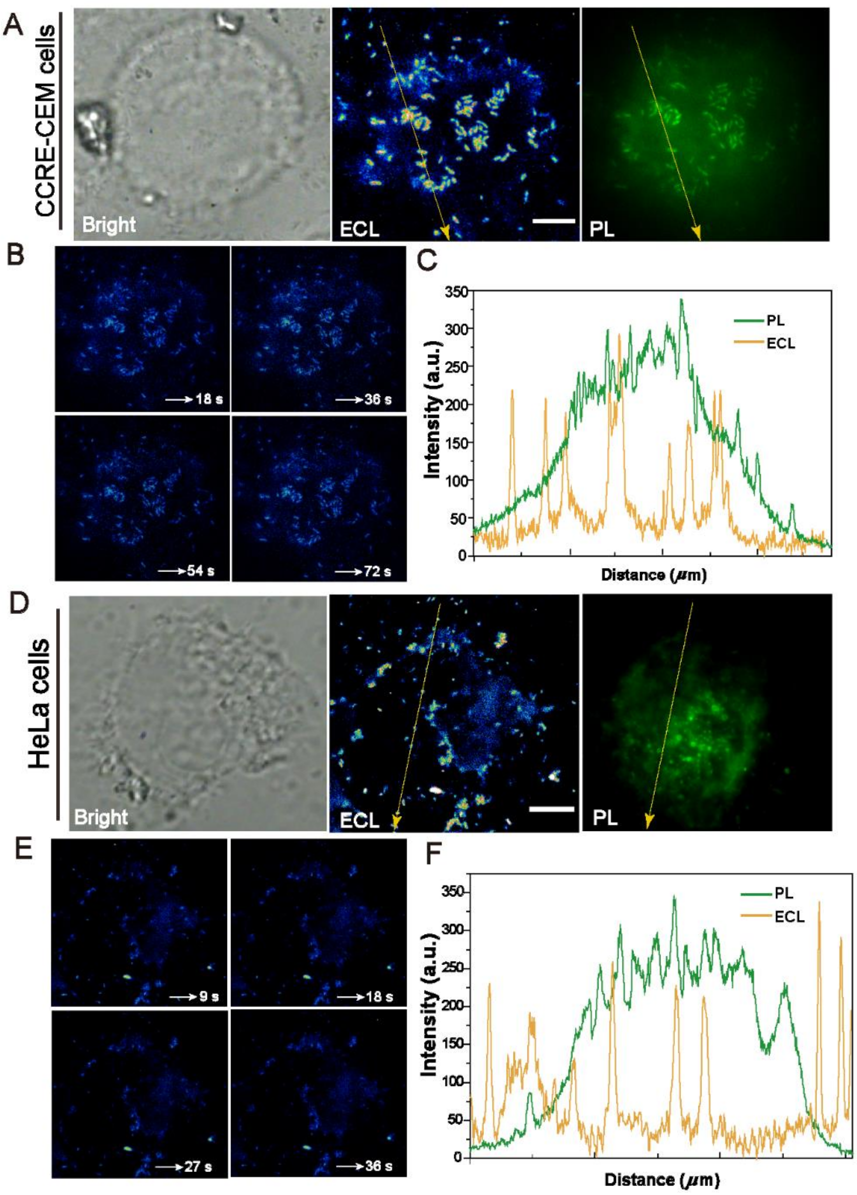

Figure S15. Characterization of the optical performance of ECL nanoemitters on living cells. (A) Bright-field, ECL, and PL images of CCRF-CEM cells. (B) Dynamic ECL imaging of CCRE-CEM cells at different moments. (C) PL and ECL spatial-intensity profiles of CCRE-CEM cells along the yellow arrow in PL and ECL image of (A). (D) Bright-field, ECL, and PL image of HeLa cells. (E) Dynamic ECL imaging of HeLa cells at different moments. (F) PL and ECL spatial-intensity profiles of HeLa cells along the yellow arrow in PL and ECL image of (D). Scale bars, 3 μm and 6 μm respectively.

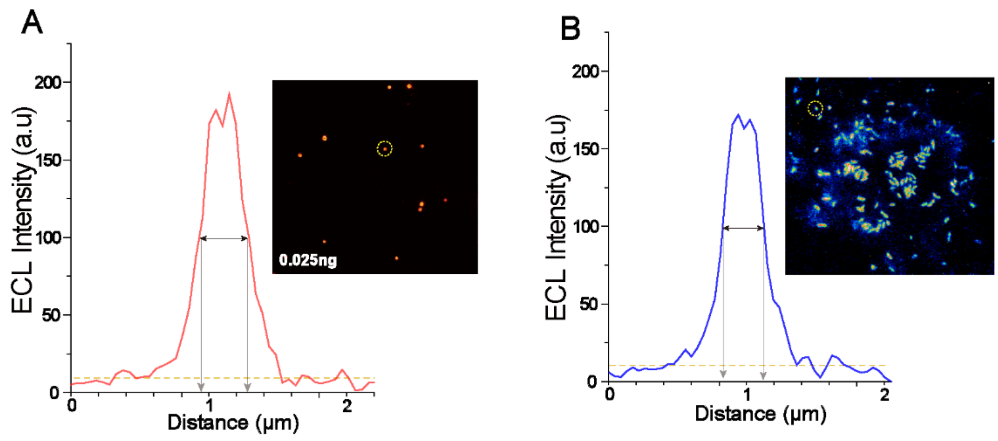

Figure S16. Validation of single protein signals on the surface of living cells. (A) The ECL imaging spot size and peak intensity from the marked signal spot in the illustration, the illustration is from **Figure S13 D**, the single protein ECL imaging experiment. (B) The ECL imaging spot size and peak intensity from the marked signal spot in the illustration, the illustration is from **Figure 5D**, the ECL dynamic monitoring of membrane proteins.

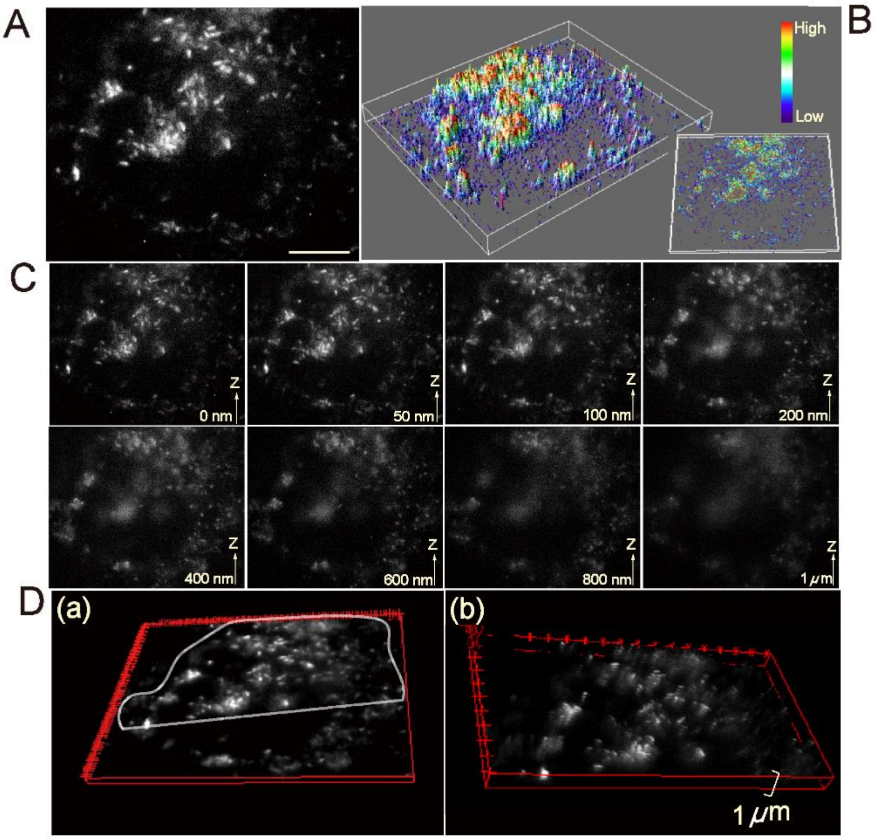

Figure S17. Characterization of the imaging performance of ECL nanoemitters on living cells. (A) ECL image of HeLa cells. (B) The 3D concentration distribution of PTK7 protein obtained by Matlab calculation program based on (C), the illustration shows a top view. (C) Visualized dynamic imaging of cells at different levels in the Z-axis direction in the same region as (A). (D) Z-stack ECL images of HeLa cells from (C), and b) is the selected regions in a). Scale bars is 4 μm.

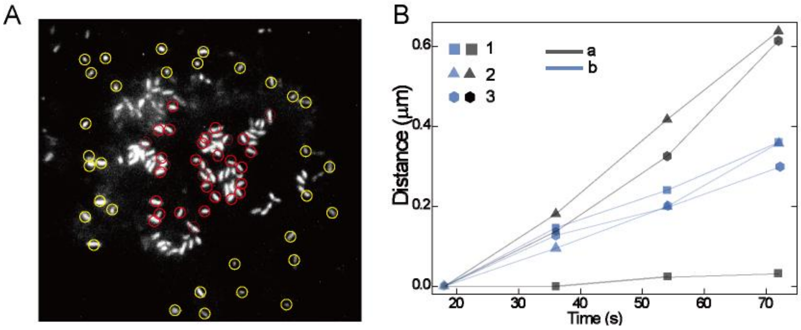

Figure S18. (A) Statistical analysis of proteins in different regions on single cell, yellow circles represent the cell peripheral proteins and red circles represent cell medial proteins. (B) Temporal evolution record of protein individuals, region **a** (gray) and region **b** (blue) in Figure 5A.

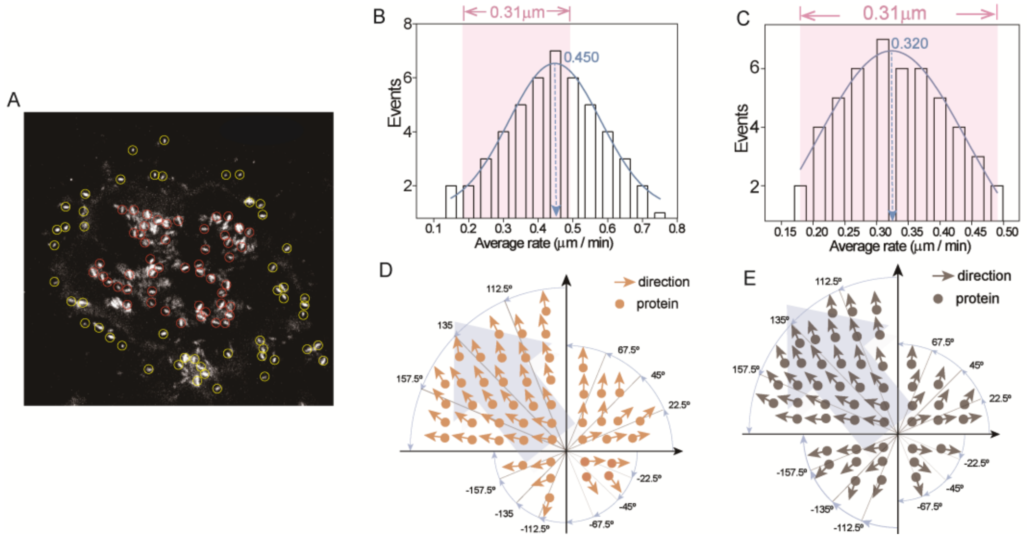

Figure S19. Dynamic analysis of protein individuals in different regions of HeLa cells. (A) Statistical analysis of proteins in different regions on single cell, yellow circles represent the cell peripheral proteins and red circles represent cell medial proteins. (B) Statistical analysis of cellular peripheral protein velocity on single cell. (C) Statistical analysis of cellular medial protein velocity on single cell. (D) Statistical analysis of the proteins movement direction of cellular peripheral region. (E) Statistical analysis of the proteins movement direction of cellular medial region.

References

[1] a) R. Chen, J.F. Zhang, J. Chelora, Y. Xiong, S.V. Kershaw, F. K. Li, P-K. Lo, K. W. Cheah, A. L. Rogach, J. A. Zapien, C. S. Lee, C.-S, *ACS Appl Mater Interfaces.* **2017**, *9*, 5699-5708; b) H. Xu, C. C Liao, Y. Liu, B. C. Ye, B.H. Liu, *Anal. Chem.* **2018**, *90*, 4438-4444.
[2] M. J, Katz, Z.J. Brown, Y.J. Colon, P. W. Siu, K. A. Scheidt, R. Q. Snurr, J. T. Hupp, O. K. Farha, *Chem. Commun.* **2013***, 49*, 9449-9451.
[3] Y. Liu, H. Zhang, B. Li, J. Liu, D. Jiang, B. Liu, N. Sojic, *J. Am. Chem. Soc.* **2021**, *143*, 17910–17914.
[4] M. Sentic, M. Milutinovic, F. Kanoufi, D. Manojlovic, S. Arbault, N. Sojic, *Chem. Sci.* **2014**, *5*, 2568-2572.
[5] G. Zhou, M. Li, *Adv Mater* **2022**, 2200871.
[6] C. Cui, R. Jin, D. Jiang, J. Zhang, J. Zhu, *Research*, **2021**, *2021*,1742919.
[7] .R. J. Millington, J. M. Quirk, *Trans. Faraday Soc.* **1961**, *57*, 1200-1207.

Author Contributions

N. Sojic, D. Jiang and B. Liu conceived and designed this study. B. Li performed the experimental work and data analysis. X. Huang and Y. Lu assisted in the experiment. Z. Fan and B. Li assisted in analyzing the data. B. Li wrote the first draft of the paper. B. Liu, D. Jiang and N. Sojic revised the paper. B. Liu, D. Jiang and N. Sojic supervised all aspects of the work. All the authors were involved in the work.
